# Addresses

**Published:** 1869-01

**Authors:** 


ADDRESSES.—The Annual Address before the Ohio State Medical Society, by the retiring President, Prof. E. B. Stevens, M. D., of Cincinnati, is before us. He describes the character and acquirements of the "good physician," and explains what he calls the "unity of his art." The address is interesting, instructive, and in the highest sense *eloquent.* We have also received Dr. Stevens' introductory address to the ninth annual course of lectures in the Miami Medical College. Better selected or more elegantly expressed advice cannot be found in the whole literature of introductory addresses.

We have received the Annual Address before the Medical Society of the State of New York, February 5th, 1868, by John P. Gray, M. D., President of the Society and Medical Superintendent of the New York State Lunatic Asylum, Utica. The object of the address is to show the connection between psycological medicine and the whole body of medical knowledge to which it belongs. He says that it was for a long time supposed that the treatment of the so-called diseases of the mind was quite a different thing from the treatment of diseases of the body; and this idea has not yet wholly disappeared. He goes on to show the connection between psycological and general medicine, relating facts which prove insanity to be subject to the same laws, and to present the same general pathological conditions which are found in other diseases. The subject is a highly interesting one, and is treated in a most satisfactory and convincing manner. If we had space we should be glad to republish the whole address, and should make room for it, only we know that many of our readers will receive it in the Transactions of the Medical Society of the State of New York.

Introductory Address of Theophalus Parvin, M. D., before the class of the Medical College of Ohio. Prof. Parvin discourses earnestly and eloquently upon the "Subjective Utility of Medicine." He quotes Abernethy of St. Bartholomew's Hospital, who, on entering the room crowded with young men just commencing their professional studies, involuntarily exclaimed, "God pity you all," and goes on to draw truthful pictures of professional life, such as all students should consider before engaging in the study of medicine.

